# Use of cannabis to manage symptoms of mental and physical health conditions during pregnancy: analysis of a pro-cannabis pregnancy forum

**DOI:** 10.3389/fpsyt.2024.1478505

**Published:** 2024-12-10

**Authors:** Rachel L. Gunn, Elizabeth R. Aston, Lia Artis, Jacqueline Nesi, Eric R. Pedersen, Lauren Micalizzi

**Affiliations:** ^1^ Center for Alcohol and Addiction Studies, School of Public Health, Brown University, Providence, RI, United States; ^2^ Department of Psychiatry and Human Behavior, Alpert Medical School of Brown University, Providence, RI, United States; ^3^ Department of Psychiatry and Behavioral Sciences, Keck School of Medicine, University of Southern California, Los Angeles, CA, United States

**Keywords:** prenatal cannabis use, symptom management, online forum, qualitative analysis, maternal health

## Abstract

**Introduction:**

Rates of prenatal cannabis use (PCU) have increased in recent years. Despite evidence of developmental health consequences to offspring and birthing person, there has been a reduction in the perception of PCU-related harms. Due to the stigma and risk of legal consequences associated with disclosing PCU, individuals are often cautious to seek information from their healthcare providers. Thus, pregnant people are more likely to seek information from anonymous sources, such as online support forums. Information garnered from these anonymous online forums can shed light on the patterns and motives for cannabis use among this population. These insights can help to better inform prevention efforts aimed at reducing potential harms of PCU and improve intervention efforts.

**Methods:**

Posts (N = 120) from an online pro-cannabis pregnancy forum called “Ganja Mamas” on WhattoExpect.com were randomly selected and analyzed if they covered topics related to PCU. A qualitative coding structure based on the existing PCU literature was created and refined to include other emergent topics. The coding structure was used to apply thematic analysis to posts; associated codes were grouped into themes. Codes specific to symptom management for physical and mental health were subsequently subjected to further conceptual analysis for the current study.

**Results:**

Four themes related to symptom management during pregnancy were identified: 1) cannabis use and impacts of use for a variety of mental health symptoms, including depression and anxiety; 2) cannabis use for physical health symptoms and conditions, such as nausea and pain; 3) use of cannabis to achieve homeostasis and manage stress; 4) decision-making about using cannabis for symptom management, such as using cannabis instead of prescription medications. Most discussions in this pro-cannabis forum reflected perceptions that cannabis was effective in treating the conditions for which it was used; however, limitations of cannabis’ efficacy were also mentioned.

**Discussion:**

There is need for reduced stigma and open communication between pregnant persons who use cannabis and their providers in discussing how to manage their mental and physical health symptoms. Understanding the various symptoms for which individuals use cannabis during pregnancy to self-treat can inform these conversations and the expansion of harm reduction strategies.

## 1 Introduction

The prevalence of cannabis use is increasing, reflecting evolving legalization policies, greater availability, shifting attitudes, and a growing recognition of potential therapeutic (1)properties of cannabis products (1–3). Prenatal cannabis use (PCU) parallels increases in the general population prevalence (4–6) with current rates reported as high as 9% in 2022 (7). PCU rates are increasing (5, 8) despite evidence that PCU may be associated with adverse outcomes for both the pregnant person and their developing child (9) and direct recommendations from the American College of Obstetricians and Gynecologists (10) and the American Academy of Pediatrics (11) to refrain from PCU Specifically, PCU is associated with increased risk of adverse outcomes for the birthing person (e.g., preeclampsia, preterm delivery), the neonate (e.g., respiratory distress syndrome, gestational size, low birthweight, and neonatal intensive care admission; (12), and childhood developmental outcomes, such as neurodevelopment and psychopathology (13, 14). As cannabis use becomes increasingly normalized, both in pregnancy and during lactation (15, 16), it is essential to understand motives for use and address the needs and concerns of individuals who use cannabis while minimizing potential harms.

Individuals also report the use of cannabis for managing various physical and mental health conditions or to improve overall well-being. The perception that cannabis is a panacea for treating a variety of symptoms has proliferated in recent years, fueled by both anecdotal accounts and emerging scientific research (17–19). Pregnant people primarily report cannabis use motives related to symptom management of chronic physical conditions that exist prior to and continue in pregnancy, for pregnancy-related physical conditions, and for improving mental health (20). In fact, individuals who continue to use cannabis after pregnancy recognition report using specifically for physical and mental health symptom management (21). Understanding these symptom management motives is of critical importance, especially given hesitancy of pregnant persons to disclose cannabis use to their providers.

### 1.1 Physical symptom management

#### 1.1.1 Nausea, vomiting, and appetite stimulation

One of the most cited motives for PCU is antiemetic relief (i.e., to alleviate pregnancy-related nausea and vomiting), which is estimated to impact 70-80% of pregnant persons (22). Moreover, findings from a study of almost 300,000 pregnancies in California found that the prevalence of PCU was greater among pregnant people with severe (11.3%) and mild (8.4%) nausea and vomiting in pregnancy compared to no nausea and vomiting in pregnancy (4.5%) (23). Antiemetic motives also appear to be tied to the persistence of PCU, as a study of cannabis use patterns found that of those individuals who continued to use cannabis during pregnancy, 96% reported use for treatment of nausea. For those who quit during pregnancy, 31% reported they used cannabis early during pregnancy to treat nausea (24), when it is most commonly experienced. A qualitative study of motives for PCU found that nearly all participants interviewed cited use of cannabis to manage pregnancy-related nausea and vomiting, as well as a perception that cannabis was more effective than pharmaceuticals (20). This finding has been replicated in quantitative research on hyperemesis gravidarum (HG), an extreme version of nausea and vomiting in pregnancy (25). Similarly, cannabis is often used to stimulate appetite. An evaluation of Pregnancy Risk Assessment Monitoring System data from eight states found that pregnant people report using cannabis “to increase appetite,” “to be able to eat,” and “to gain weight” (26).

#### 1.1.2 Pain

Pregnant individuals may also use cannabis to manage pain that onsets during pregnancy, as well as for chronic pain conditions that existed before pregnancy and are exacerbated by the physical changes and hormonal fluctuations that occur during pregnancy (20). Qualitative research has found that pregnant people use cannabis to “help with” and “get past” pain (27). Moreover, a cross-sectional survey (*N*=1,749) found that second to depression and anxiety (63%), help with pain (60%) was the most reported motive for cannabis use in pregnancy (28). Outside of pain and antiemetic/appetite motives for PCU, less is known about the range of physical conditions for which pregnant individuals may use cannabis.

### 1.2 Mental health symptom management and stress relief

Anxiety and depression are common mental health conditions in pregnancy and those who use cannabis in pregnancy are more likely to have a history of depressed mood and/or anxiety (29–33). For example, data from the National Survey on Drug Use and Health, indicated that past 30-day cannabis use was more prevalent among pregnant persons that had depression versus those that did not [12.7% versus 3.7% (30)]. Data from the National Epidemiologic Survey on Alcohol and Related Conditions similarly found that the odds of cannabis use and cannabis use disorder were greater for those with any past-year mood, anxiety, or posttraumatic stress disorders (29). Pregnant people with high depression scores are also at increased risk of continuing to use cannabis throughout pregnancy (34). While causal claims cannot be drawn from these data, many pregnant individuals may report subjective improvements in mood or anxiety after using cannabis. Direct report from individuals who are using cannabis during pregnancy can help shed light on the motives and impact of this use for both physical and mental health conditions.

### 1.3 Opportunities in qualitative research and discussion boards

Despite recreational and medical legalization in many U.S. states, there is still stigma around use of cannabis, especially for pregnant people (35). Pregnant people are thus hesitant to disclose cannabis use to their providers (36) and therefore may miss out on important health information bestowed by providers. In addition, patients who use cannabis may also perceive stigma directly from providers, further limiting willingness to discuss their use (37). Given this stigma in the current social climate surrounding cannabis, qualitative research on PCU from anonymous, naturally occurring conversations is invaluable for gaining a nuanced understanding of the complexities surrounding this topic, as has been shown in prior studies on parenting and maternal topics (38–40). Public forums on websites are common venues for pregnant people to converse with peers about pregnancy-related topics (41–44). These forums attract individuals who provide a rich array of perspectives on the topic, including insights into the motivations behind cannabis use, perceived benefits, and potential risks. Moreover, the naturalistic environment of these forums may support anonymity relative to other qualitative observational research, as they encourage participants to express themselves freely, often providing candid and unfiltered accounts of their experiences. Discussion boards also facilitate interaction and dialogue among participants. This structure can elicit authentic and spontaneous responses, and reveal social dynamics, providing researchers with insights into how people naturally discuss PCU.

As it relates to PCU, attention has been brought to one discussion forum in particular. “Ganja Mamas” on *WhattoExpect.com* is branded as a forum for “Ganja Mamas looking for other supportive ganja mamas.” A study of 1260 comments posted to the Ganja Mamas forum over a 7-day period in December 2020 (42) found that individuals in this forum exchange geographically specific information, particularly related to drug testing and Child Protective Services (CPS), and that relief and reassurance are expressed in experiential dialogues. Another study of Ganja Mamas content from June 2020 to May 2021 identified several themes related to the perceived impact of cannabis use in pregnancy on the developing child (43).

### 1.4 Current study

The goal of the current study was to complement these studies by exploring how individuals who engage with this pro-cannabis forum discuss the use of cannabis for physical and mental symptom health management during pregnancy. A qualitative analysis was conducted on posts from Ganja Mamas that discussed cannabis use for mental and physical symptoms. The goal was to identify themes present in these posts to better understand the range and extent of symptom management motives and outcomes of PCU.

## 2 Materials and methods

### 2.1 Qualitative data preparation

Discussion threads posted between June 2020 and May 2021 were extracted from the pregnancy and parenting cannabis use sub-forum “Ganja Mamas” on *WhattoExpect.com*. Threads were structured by a topic statement, often a question or general idea posted by a forum member, and any number of responses or comments from other members. Thread discussions generally follow the format of answering questions posed by others or sharing similar experiences to the original poster. Ten threads in each month (*N*=120) were selected using a randomization function within a python script. A deductive approach to coding was utilized in this investigation (45). See 43 for a full description of the study methods. Posts were determined to be appropriate for subsequent analysis if the thread included language regarding cannabis use during lactation or pregnancy; thread inclusion was deemed suitable via comprehensive post evaluation by two or more coders. Posts were determined to be inappropriate for inclusion if the thread was not relevant to use of cannabis during lactation or pregnancy (e.g., mention of cannabis use outside the context of pregnancy). Seven threads violated these inclusion criteria and were removed prior to analysis, resulting in 113 threads for analysis.

### 2.2 Data analysis

A qualitative coding structure was created following an initial review of the literature pertaining to PCU. This coding structure was updated throughout the coding process to allow for inclusion of emergent topics. Threads underwent two coding cycles: descriptive (Cycle I) followed by conceptual (Cycle II). During Cycle I, threads were analyzed by two individual coders (including authors RLG, ERA, JN, LM) using applied thematic analysis (46). Threads were evaluated to identify important topics via an open coding procedure (45). Potential codes were updated as the coding procedure proceeded, and related codes were sorted and synthesized to build themes. Coders convened weekly to attain consensus and resolve any discrepant codes. Coder adjudication, simple coder consensus, and intensive coder discussion were employed to resolve such discrepancies and reach agreement (47–50). Final codes were uploaded to *NVivo* (51) qualitative data analysis software to enable organization and synthesis of codes. Following completion of the initial open-coding evaluation, Cycle II coding was initiated. Codes and sub-codes relevant to using cannabis to manage physical or mental health symptoms were assessed and subsequently underwent a second cycle of coding focused on classification, integration, synthesis, and conceptualization of topics (52). Quotes were then summarized individually by two coders to identify prominent themes. Coders achieved thematic consensus and chose representative quotes to illustrate each theme (see Supplementary Figure 1 in 43). Consistent with acceptable methods for qualitative data presentation, topics and themes are described but not quantified as data obtained from forum threads would not accurately represent the frequency of a particular belief or behavior (53).

### 2.3 Confidentiality and data protections

Qualitative data were obtained from a public forum with no requisite registration for access; authors did not participate in forum discussions. User identities are anonymous on the forum, identified by usernames only. No identifiable information was collected. As such, this investigation did not meet the definition of research with “human subjects” and our local Institutional Review Board deemed that it was not subject to review. Further precautions to preserve group anonymity were taken, primarily omission of usernames of contributors in this manuscript. All quotes included in this manuscript were adjusted by altering text slightly to further preserve the anonymity of posters (48). Retention of the original language verbatim would permit quotes and usernames to be searched via internet search engines. Consequently, slight alteration of the language, while retaining meaning and sentiment, was critical and is typical in the field (48, 54, 55), including in prior work by our group (43). Decisions pertaining to data acquisition and analysis were informed by ethics laid out in previous work (56, 57).

## 3 Results

Four themes related to symptom management were identified: 1) cannabis use for mental health symptoms and conditions; 2) cannabis use for physical health symptoms and conditions; 3) use of cannabis to achieve homeostasis and manage stress; and 4) decisions about using cannabis for symptom management.

### 3.1 Use of cannabis for mental health symptoms

Posters discussed using cannabis to manage various mental health symptoms and related problems. Some of these comments were general in nature, for example, one person discussed using cannabis for their mental health broadly, stating

“*I’ve continued to smoke the whole time even though I’m worried, because it helps so much with my mental health*.”

Another stated,

“*quitting is too hard and I wouldn’t know what to do because I am constantly sick when I am not smoking and use it for mental health.”*

However, most comments referenced how cannabis helped them to manage specific mental health symptoms and conditions, such as anxiety and depression.

#### 3.1.1 Anxiety

Posters reported that cannabis helped to manage anxiety both generally (i.e., outside of the context of pregnancy), as well as anxiety that is specific to pregnancy. One person stated,

“*I’ve been using marijuana before getting pregnant for my anxiety, but now I’m even more anxious while pregnant … and smoking even more*.”

Similarly, another forum member noted how effective cannabis use was for managing their anxiety post-delivery, explaining

“*it helps me so much, it’s natural. I use it for my PPA [postpartum anxiety].*”

All comments related to using cannabis for anxiety management suggested that cannabis was a helpful tool (i.e., “*calms me”* or “*significantly lowers my anxiety*”). One member stated they are looking forward to quitting cannabis, but noted it was difficult due to anxiety, commenting

“*really looking forward to quitting, but it is really hard with bad anxiety!!”*

#### 3.1.2 Depression

Posts reflected on use of cannabis to manage symptoms of depression. One individual noted that cannabis helps improve their mood,

“*my partner often notices, I could be irritable/sad and as soon as I smoke I am in an good mood, I become kind, and much more loving in general.”*

Another individual noted cannabis helps improve their energy, explaining that

“*smoking weed has made me more energetic and more alert as a new mom*.”

While most comments reported cannabis helped to improve mood, one poster noted,

“*I’ve tried to smoke and it didn’t help. I want to feel better. I’ve never felt so terrible in my life and I honestly get so depressed without it,”*

suggesting cannabis did not significantly improve their depressed mood, but prevented their mood from worsening.

#### 3.1.3 Sleep and insomnia

Sleep was another common reason for reporting cannabis use during pregnancy. For instance, one forum member commented,

“*I had around 5mg last night and slept good for the first time in a while!”*

Another person noted they did not use cannabis regularly until pregnancy when they began using for sleep, explaining

“*im 18 weeks and I never liked smoking until a week ago when it helped me sleep.”*

One poster also provided advice regarding using cannabis for sleep, instructing other forum members to

“*start with 1 or 2 mg, especially if it’s just to help sleep.”*

#### 3.1.4 Other mental health symptoms

The use of cannabis to manage symptoms associated with other mental health conditions was also discussed, but often in combination with anxiety and depression. One poster noted that cannabis was used for their symptoms of post-traumatic stress disorder (PTSD), stating

“*I smoke weed for my anxiety, depression, and PTSD*.”

Another poster reported that they used cannabis to help increase their interest in sexual activity, for instance, one poster described,

“…*I have totally lost my sex drive and my partner is starting to think I’m not attracted to them anymore. With Valentine’s Day coming up, they planned a really romantic day and night for us and I really want to have sex afterward … I know if I have a couple hits off my bowl it’ll get me in the mood (cannabis does that for me). Please give me some advice.”*

In addition, one comment referred to the use of cannabis to deal with symptoms that are related to attention-deficit hyperactivity disorder (ADHD), stating

“*I suffer from ADHD triggered anxiety. My doctor has given me approval to stay on ADHD meds this pregnancy … last pregnancy i came off of them and did not smoke (it was my first) i was miserable completely totally miserable I’m talking i was sick the entire time and i was depressed due to my ADHD and off meds while trying to do my very demanding job.”*

### 3.2 Use of cannabis to manage physical health symptoms

Members of the forum also commented on their use of cannabis to manage a variety of physical health symptoms.

#### 3.2.1 Gastrointestinal symptoms, appetite, and weight gain

A variety of gastrointestinal symptoms, including appetite and weight, were a common topic of conversation for posters. Posters discussed how cannabis was helpful for managing symptoms related to irritable bowel syndrome and for improving bowel movements, commenting

“*it’s helping enormously with EVERYTHING like bowel regularity*.”

Posters also commented on how cannabis helped improve appetite, noting that it

*“helps enormously with my appetite.”*

Similarly, another poster commented that their appetite worsened when reducing CU, explaining

“*I had tried to stop but my morning sickness was extreme and for the entire day and I would have no appetite.*”

Many members noted the use of cannabis to help with weight gain related to loss of appetite. One forum member discussed that they were,

“*very worried and freaking out at the start of my pregnancy because I tried to stop smoking many times but I’ve smoked for six years I was just losing weight and getting so sick.”*

While most comments stated that cannabis helped with appetite, one poster noted it was not effective, stating

“*I have absolutely ZERO appetite even when I’m high.*”

#### 3.2.2 Nausea/morning sickness

In addition to managing appetite and weight gain, members often agreed that cannabis helps to manage symptoms of nausea and morning sickness. For example, one person said they,

*“had terrible morning sickness throughout pregnancy, marijuana helped.”*

Another poster noted that cannabis helped to reduce vomiting during pregnancy, commenting

“*I smoked a lot … I didn’t limit myself at all because … it was all I could do to eat without throwing up.*”

One person explained that they used additional pharmacological treatment for nausea, but that they used cannabis to be able to swallow those medications, explaining

*“I have to smoke just so I can keep down nausea meds.”*

The amount of cannabis needed to abate nausea was often discussed; for example, one member noted

“*some days I’ve gone through 15 grams if I’m puking too much and can’t eat and I only smoke the strong stuff … I’m very very particular on the type of weed I smoke*”.

Posters also discussed conducting risk-benefit analysis when it came to choosing cannabis to treat nausea, noting

“*my husband and I decided that being able to eat and our baby getting food was worth the risk.”*

Another member of the forum explained that their healthcare provider suggested that cannabis may itself lead to increased nausea, stating

“*I went to the emergency room this week and the doctor came back and rudely told me to stop smoking. He said the weed may be causing the nausea.*”

In addition to comments about nausea and morning sickness, there were several references to using cannabis to manage HG. For instance, one poster noted that they were,

“…*diagnosed with HG and prescribed a cocktail of nausea meds. Promethazine pills? and suppositories along with Zofran. The combo does give some relief. But I still get sick and can’t eat. The only thing that provides relief is cannabis. It gives me the energy to get up so I can eat and I’m not nauseous.”*

Several posters felt that cannabis was the preferred way to manage their HG after trying several other treatments, explaining

“*I thought [HG] would end after first trimester but looks like it is continuing … I’ve been in the hospital several times, no medications help, had to switch from orals to iv infusions to pessaries and still non worked except for weed*.”

In addition to HG, another person commented that cannabis helped manage symptoms related to their Crohn’s disease, discussing

“*I used cannabis frequently throughout my pregnancy because I have Crohn’s disease and it keeps me in remission.”*

#### 3.2.3 Pain

There were references to cannabis use for both general pain and for specific experiences of pain. For example, one poster commented,

“*I am pregnant and if I didn’t smoke marijuana I would be in debilitating pain.*

Another posted that cannabis helped with pain related to Fibromyalgia, stating

“*I have fibromyalgia and smoking works so much better than the prescription meds.”*

One poster commented on using cannabis for endometriosis, explaining

“*My endometriosis is painful, so I smoke for that*”.

cannabis use was also deemed helpful for back pain, with one forum member noting

“*my back pain is horrible and … smoking helps me so much.*”

Others reported using to help with headaches or migraines, explaining

“*I use weed as much as I need to treat migraines, including micro dosing (less than 10 mg) daily with edibles.”*

Still others discussed use to aid in the alleviation of “…*neuropathy”.* Some commenters mentioned their plans to use cannabis to manage labor pain, with one individual specifying plans to vaporize cannabidiol (CBD), a non-psychoactive cannabinoid,

“*for pain during labor so I’ve been researching which ones have high cbd content/are best.”*

### 3.3 Achieving homeostasis/stress management

Another theme that was identified was the use of cannabis for managing stress and to reach a state of homeostasis. For instance, one poster commented,

“*I hit a bowl once a day just to be evened out.”*

In the context of using cannabis to manage stress, another poster commented,

*“…it’s been heaven sent for me … i firmly believe it’s medicine and that it’s better to be less stressed…*”

and yet another noted,

“*smoking helps me chill and unwind, and basically have faith that everything will all work out.”*

*cannabis use* was reportedly helpful for handling daily stress related to parenting and career management, explaining

“*it’s been the cure to my post partum problems and daily stress as a full time parent with a demanding career and smart beautiful toddler.”*

### 3.4 Decisions about using cannabis for symptom management

In addition to referencing the symptoms that group members use cannabis to manage, they also discussed the decision-making that surrounded choices to use cannabis for symptom management. For instance, several posters discussed the preference for cannabis over prescription medications for various conditions and for perceiving stigma from others about their CU, with one forum member stating,

“*I do not believe any medicine, prescribed or over the counter, is better to you and baby than cannabis.”*

Another forum member stated,

“*I don’t see how they find it medically suitable to prescribe promethazine with all of the awful side effects it causes, but judge cannabis (a plant from the earth).”*

Members also discussed their medical cannabis use broadly, explaining

“*I use marijuana as medicine*,”

while another person referenced continued medicinal cannabis use across the postpartum transition, stating,

“*I use marijuana as medicine and did so throughout my pregnancy and I’m still breastfeeding my 22 month old.”*

Posters also sought advice from the forum when not using cannabis because of pregnancy, with one individual asking

“*any tips on dealing with the anxiety? I already suffer from that and depression, but I am not taking any medication. Ganja was my medication anything can help!”*

## 4 Discussion

The purpose of this investigation was to examine posts related to using cannabis for the management of mental and physical health symptoms from a public pro-cannabis forum on a popular pregnancy and parenting site. Posters discussed a range of symptoms that they used cannabis to manage, reflected the positive impact of cannabis for treating these symptoms, and shed light on their decision-making around PCU. Topics discussed also included themes of harm reduction (i.e., cannabis as less harmful than specific medications or the impact of the symptoms themselves). This research builds on our prior work (43) and the work of others (42) suggesting these forums are used to cover a wide range of topics related to cannabis use among perinatal individuals. An understanding of specific symptom-management motives increases our understanding of the symptoms that people use cannabis to manage and informs the development of effective treatments that both reduce risk and address the needs of peripartum people.

Consistent with prior literature (23, 58), cannabis use for antiemetic reasons and for increasing appetite was frequently referenced as a motive for PCU. Forum members generally discussed how cannabis was helpful for increasing appetite, reducing symptoms of nausea and vomiting, and weight gain. In general, when discussing nausea, vomiting, and appetite, several posters reflected their experience using prescription and non-prescription remedies without any improvement in symptoms, and that cannabis provided more relief than these other treatments. Several posters specifically discussed HG, and shared that cannabis was the only thing that would alleviate their severe symptoms (e.g., dehydration requiring hospitalization). This pattern of use is consistent with quantitative literature suggesting that pregnant people with HG use cannabis when other treatments are ineffective (25). In the wake of initial investigations into cannabis use to treat HG (59), there is concern in the medical community (60, 61) that those using cannabis for HG may in fact be experiencing undiagnosed cannabinoid hyperemesis syndrome (severe and chronic vomiting from prolonged cannabis use). In fact, one poster may have described an experience in which their provider suggested this possibility during a medical visit. In general, and consistent with use of cannabis to treat other symptoms, posters discussed the perception that cannabis was less harmful to their developing child than the symptoms they were experiencing, motivating their decision to use cannabis to provide relief.

The perception that cannabis was the only effective treatment, relative to other prescription and non-prescription medications, was consistent in posts about sleep and pain as well. Despite mixed evidence on the association between sleep and cannabis use (62), members discussed how cannabis helped them sleep. These reflections were shared both by those who used cannabis prior to pregnancy, as well as those who initiated cannabis use in pregnancy due to sleep difficulties. Those who use cannabis during pregnancy may not actually see improvements in sleep (63), suggesting this may be increasing cannabis-related risk without the anticipated benefits. Regarding pain, there was consistent discussion of the effectiveness of cannabis use for managing pain from a variety of sources. While the research on pain is diverse and the efficacy of cannabis use for pain relief is beyond the scope of this investigation [see review articles (64–66)], it is likely that individuals who are pregnant and experiencing pain, receive some relief, at least acutely (67), from cannabis. Overall, given the consistent self-report of cannabis as effective for a variety of physical conditions in this analysis, and paired with the consistent sub-theme that cannabis is perceived as more effective than traditional treatments, it is critical for researchers and providers to engage in expanded research and open conversation to better understand alternative treatment for these conditions.

Posters consistently reported cannabis as helpful for a variety of mental health conditions including depression, anxiety, ADHD, and PTSD. Posters also discussed the use of cannabis for general stress management and to achieve homeostasis and the preference for cannabis over traditional prescription or non-prescription (i.e., over the counter) medications, consistent with prior qualitative work in medical cannabis card holders (68). Also consistent with self-treatment of physical conditions with cannabis and our prior investigations (43), members discussed cannabis use as a harm reduction strategy for a variety of conditions and symptoms. Often, they also discussed how, despite advice given from their medical providers, they believed cannabis to be safer than other treatments, especially prescription medications. Although some prescription medications have been found to be safe in pregnancy (69), and given the variety of effective behavioral and cognitive-based treatments for treating mental health even specifically during the perinatal period (70, 71), there is a clear need to reach individuals during pregnancy to provide education and access to these safe and effective treatments. Overall, when discussing the decision to use cannabis to manage symptoms, posters mentioned the belief that cannabis either worked better than other traditional treatments or was safer than traditional treatments or experiencing the symptoms themselves. This theme was consistent across discussion of both mental and physical health symptoms.

Interesting, many of the posts referred to “smoking” cannabis as opposed to other variations of product use, such as cannabis-infused edibles or vaping devices. There is evidence that smoking cannabis can have harmful effects, particularly on the respiratory system of the smoker (72). However, we did not observe much cautionary advice around limiting smoking or promotion of other forms of cannabis intake. Data on these forums were collected in 2020 and 2021, prior to the burgeoning of the legal recreational market in many states that helped to increase accessibility to cannabis edibles, topicals, tinctures, and vapes. Updated contemporary analyses of cannabis pregnancy forums will be important to learn more about the perceived benefits and risks of alternate forms of cannabis use beyond smoking.

### 4.1 Limitations

This study analyzed posts to a public forum to better understand PCU symptom management motives. While there are strengths to this approach (i.e., data that captures the naturally occurring social discussion around PCU without bias introduced by human subjects’ research), it also presents limitations. First, we are unable to verify the pregnancy status or cannabis use of the posters. Although there seems to be little motivation to falsify pregnancy in a forum like this, we were not able to verify that each post was from a pregnant person using cannabis. Similarly, because of the anonymity of the forum, we are not able to provide demographic information or other characteristics of the sample. Related, we were unable to measure other potential variables that are critical to the etiology of PCU, such as experience of adverse childhood experiences, which have been shown to predict health problems among those who use cannabis during pregnancy (73). Second, these posts are collected from a pro-cannabis forum, therefore opinions of the population at large cannot be derived from this sample who self-selected to post in a forum that is marketed to specifically “support ganja mamas.” Further, there may be additional bias among those who tend to seek information from online forums (e.g., those resourced enough to have internet access). Third, the random selection of forum posts analyzed here were posted during the COVID-19 pandemic, which may have impacted forum members’ opinions on cannabis use or mental health during a period of social isolation. Future work should examine the symptom management motives of pregnant individuals with a range of experience with cannabis (e.g., those who are recently abstinent versus those who continued using in the prenatal period) to better characterize and understand the symptoms that most strongly motivate cannabis use and the individuals who are most likely to use cannabis during pregnancy.

## 5 Conclusion

This study provides a snapshot of the symptom management motives for PCU. We found that individuals discuss using cannabis during pregnancy for a wide range of both physical and mental health concerns and that largely, at least among individuals in this pro-cannabis forum, cannabis is seen as highly effective and low risk. In the wake of increased prevalence and medicalization of cannabis use, and specifically PCU, there is a pressing need to understand the key determinants of use and offer safe and evidence-based treatments to pregnant people. The hesitation to openly discuss cannabis use between providers and patients creates a challenging space for pregnant people to make decisions about their healthcare and substance use and leads to the use of anonymous online forums where information may be less reliable or accurate. More evidence-based research, open communication, training, and tools are needed to treat the conditions for which individuals use cannabis to treat.

## Data Availability

The original contributions presented in the study are included in the article/supplementary material. Further inquiries can be directed to the corresponding author.
